# Prenatal alcohol exposure induces anxiety and depressive-like behaviors with deficits in growth and food intake in mice

**DOI:** 10.3389/fcell.2026.1742806

**Published:** 2026-02-24

**Authors:** Kamal Smimih, Bilal El-Mansoury, Mohamed Marghich, Chaima Azzouhri, Nadia Zouhairi, Mustapha Agnaou, Morad Guennouni, Naima Fdil, Abdelali Bitar, Mahmoud M. A. Abulmeaty, Dara Aldisi, Qutaibah Oudat, Mourad A. M. Aboul-Soud, Mohamed Merzouki, Omar El Hiba

**Affiliations:** 1 Biological Engineering Laboratory, Faculty of Sciences and Technology (FST), Sultan Moulay Slimane University, Beni Mellal, Morocco; 2 Nutritional Physiopathology, Neuroscience and Toxicology Team, Laboratory of Anthropogenic, Biotechnology, and Health, Faculty of Sciences, Chouaib Doukkali University, El Jadida, Morocco; 3 Laboratory of Aquatic Systems: Marin and continental Environment, Department of Biology, Faculty of Sciences, Ibn Zohr University, Agadir, Morocco; 4 Science and Technology Team, Higher School of Education and Training, Chouaib Doukkali University, El Jadida, Morocco; 5 Biochemistry Laboratory/Metabolic Platform, Faculty of Medicine, University Cadi Ayyad, Marrakech, Morocco; 6 Department of Community Health Sciences, College of Applied Medical Sciences, King Saud University, Riyadh, Saudi Arabia; 7 Department of Kinesiology and Public Health, California Polytechnic State University, San Luis Obispo, CA, United States; 8 Center of Excellence in Biotechnology Research, College of Applied Medical Sciences, King Saud University, Riyadh, Saudi Arabia

**Keywords:** anxiety-like behaviors, brain oxidative stress, depressive-like behaviors, food intake, growth impairment, morphometry, neurobehavioral alterations, prenatal alcohol exposure

## Abstract

**Background/Objectives:**

Prenatal alcohol exposure (PAE) has been recognized as a significant public health concern due to its consequential and long-lasting effects on the central nervous system (CNS) and the subsequent behavioral impairments in affected individuals. The current study aims to evaluate postnatal neurobehavioral disturbances, specifically mood state and potential morpho-functional changes, as well as brain oxidative stress in mice prenatally intoxicated with ethanol at the adult stage.

**Methods:**

female mice with positive vaginal plugs were divided into three groups: Group 1 (ethanol intoxicated): received ethanol at a dose of 1 g/kg (i.p.) on gestational days 10 and 13 (two injections in total), along with pyrazole (100 mg/kg by i. p.) to inhibit ethanol metabolism and simulate chronic fetal exposure. The second group received pyrazole alone at the same dose (100 mg/kg i. p.). Group 3 (controls): received physiological saline solution (NaCl 0.9%) at the same volume as both ethanol and pyrazole. Offspring pups from the intoxicated dams were subjected, at the adult stage (from postnatal days P95 to P103), to a series of morphometric, biometric, neurobehavioral, and biochemical analyses.

**Results:**

Our data show an obvious decrease in body weight and size, decreased food intake, and skeleton deformations. Additionally, PAE mice exacerbated anxiety-like and depressive-like behaviors as well as elevated brain oxidative stress.

**Conclusion:**

The current data demonstrate the powerful neurotoxic effect of prenatal ethanol exposure on neuropsychological development as well as the associated morpho-functional changes.

## 1 Introduction

Prenatal Alcohol Exposure (PAE) refers to the consumption of alcohol during pregnancy, which can have adverse effects on fetal development (Popova et al., 2016). Alcohol is a teratogenic molecule that can easily cross the placental barrier and interfere, therefore, with normal fetal development (Popova et al., 2023a), and represents one of the most common early brain insults (Brancato et al., 2018). It is well established that PAE can lead to a wide range of neurodevelopmental disorders including physical and behavioral abnormalities known as Fetal Alcohol Spectrum Disorder (FASD) (Popova et al., 2023b; Ghasoub et al., 2025). Indeed, FASD encompasses a wide range of physical, cognitive, behavioral, and neurodevelopmental impairments that can lead to irreversible impacts on the lives of affected individuals (Riley et al., 2011). It can also affect cognitive, motor, and emotional functions, and potentially leading to greater alcohol intake during adolescence (Risbud et al., 2022). To date, no safe level of PAE has been identified, and the World Health Organization (WHO) consistently recommend complete avoidance of alcohol during pregnancy (Centers for Disease Control and Prevention, 2005; Graves et al., 2020; World Health Organization, 2014; Lewis, 2009). Despite these recommendations, an estimated 10% of pregnant women globally continue to consume alcohol (Popova et al., 2018; Popova et al., 2017). The highest prevalence is observed in the European Region, where approximately 25.2% of pregnant women report alcohol use aligning with the region’s broader patterns of heavy drinking, binge consumption, and alcohol use disorders (Popova et al., 2023b). It is important to note that even low alcohol consumption during pregnancy can present risks for fetal development (Popova et al., 2017). Therefore, the severity and specific manifestations of FASD depend on the timing, amount, and pattern of alcohol consumption during pregnancy. Even more, according to genetic studies, performed on a large human cohort (Tadokoro et al., 2025; Gao et al., 2019), it appears that the genetic polymorphism of alcohol dehydrogenase (ADH) plays a critical role in worsening the ethanol effect on the liver (steatosis, hepatitis and hepatocellular carcinoma) (Gao et al., 2019; Zahid et al., 2019) and even on other human organs including the heart (Li et al., 2019). Such finding leads to consider seriously the pathological state of the pregnant woman. Therefore, several experimental studies on rodents have used either genetic (Rungratanawanich et al., 2023), or pharmacological models (particularly pyrazole as inhibitor for ADH activity) to mimic ADH deficiency, and subsequently evaluate postnatal outcomes following low-level prenatal alcohol exposure.

It is essential to raise awareness of the risks of PAE and to implement effective prevention strategies to protect the health and wellbeing of the mother and developing fetus (May et al., 2009). It is well known that the developing central nervous system (CNS) is especially susceptible to the harmful impacts of exogenous chemical contaminations, including alcohol (May et al., 2009; Alfonso-Loeches and Guerri, 2011; Abbaoui et al., 2016; Benammi et al., 2017). This poses significant risks to the fetus, potentially leading to growth issues before and after birth, as well as abnormalities in skeletal and craniofacial development and serious alterations in CNS function together with deep locomotor deficits (Smimih et al., 2023). PAE could lead to brain damage through multiple mechanisms, with one of the primary pathways involving the excessive generation of reactive oxygen species (ROS) and a concomitant disruption of the brain’s antioxidant defense systems (Brocardo et al., 2011; Gil-Mohapel et al., 2019).

Numerous animal studies have demonstrated both morphological and behavioral alterations in offspring following PAE (Molina et al., 1987; Gaztañaga et al., 2020; Molina et al., 1984). PAE has been shown to disrupt neuroendocrine function in the offspring, particularly affecting the hypothalamic-pituitary-adrenal (HPA) axis, a critical regulator of the stress response (Weinberg et al., 2008). Additionally, PAE may alter emotional regulation, especially in adolescence, and has been linked to increased susceptibility to anxiety and depressive-like behaviors (Risbud et al., 2022; Hellemans et al., 2010). While many studies have primarily focused on behavioral teratogenic effects in juvenile animals, fewer have explored the long-term consequences during adulthood.

In particular, we emphasize that, while many studies have explored the effects of PAE, few have examined the consequences of an interaction between ethanol and ADH inhibitors such as pyrazole on neurobehavioral development. Pyrazole is a competitive inhibitor of ADH, the enzyme that catalyzes the conversion of ethanol into acetaldehyde. Its administration prevents the rapid metabolism of ethanol, prolonging its bioavailability and accentuating its teratogenic effects (Cederbaum, 2012). This pharmacological strategy makes it possible to study the direct effects of ethanol in a more targeted way, independently of those of its metabolites.

In our study, the ethanol-pyrazole combination was administered during a critical developmental window; the 10th to 13th of gestation (D10 to D13), a period corresponding in mice to key stages of organogenesis and neural tube formation (Sulik et al., 1981). This period is particularly sensitive to the teratogenic effects of alcohol on the developing nervous system.

Thus, the present study aims to address this gap by evaluating anxiety and depressive-like behaviors in adult mice following intrauterine ethanol exposure, alongside an assessment of oxidative stress markers in the brain. To achieve this, we administered ethanol at a dose of 1 g/kg i. p. to mice on gestational days 10 and 13, which correspond to the development of the CNS, particularly the formation of the neural tube that is equivalent to the third–fourth week of human gestation (DeSesso et al., 1999; Rice and Barone, 2000).

## 2 Materials and methods

### 2.1 Chemicals

Ethyl alcohol (Darmstadt, Germany) and pyrazole were purchased from SIGMA-ALDRICH (St. Louis, MO 63103 United States).

### 2.2 Animals

The study was carried out exclusively on Swiss Albino mice from the central-care animal facilities of the Faculty of Science, Chouaib Doukkali University, El Jadida, Morocco. Mice were housed in Plexiglas cages and maintained under controlled conditions, including a constant ambient temperature of 25 °C, a 12-h light-dark cycle, and free access to water and food. Efforts were made to minimize stress, suffering, and distress. The welfare of the animals participating in the study was given the utmost attention, and all procedures were carried out in accordance with ethical standards outlined in the Guide for the Care and Use of Laboratory Animals by the National Research Council. In addition, the study protocol received approval from the Moroccan Ethics Committee for Animal Research (MECAR) under the reference number (MoSEAR Ref: UCD-FS-06/2024, 10 February 2024).

### 2.3 Treatments

A total of 18 virgin female mice aged 15 weeks were included in our experiments. Each litter was represented by a single male and female assigned to the same experimental condition to limit litter bias (the litter considered the biological unit). The presence of a visible vaginal plug was checked every 4 h and used as a criterion for confirmation of mating. According to standard terminology in rodent prenatal research, we considered the day of vaginal plug appearance as the first day (D0) of pregnancy.

Female mice (28 ± 4 g) with positive vaginal plugs were then divided into three groups, with six mice in each group.

✓ Group 1: (Et + Pyr): Pregnant female mice received two intraperitoneal injections of ethanol (1 g/kg) determined according to previous reports (Ukita et al., 1993), with adaptation according to our trials in mice, taking into consideration intrauterine mortality (see our previous paper (Smimih et al., 2023). The first injection is after 10 days following positive vaginal plugs (D10), while the second is on the 13th day of gestation (D13). One hour before ethanol injection, each mouse received an intraperitoneal injection of pyrazole at a dose of 100 mg/kg (prepared in 0.9% NaCl). Pyrazole is a competitive inhibitor of ADH, which contributes to the conversion of ethanol into acetaldehyde, used to prolong ethanol bioavailability and accentuates its teratogenic effects (Brocardo et al., 2011). One hour before ethanol injection, each mouse received an intraperitoneal injection of pyrazole at a dose of 100 mg/kg (prepared in 0.9% NaCl).
✓ Group 2 (Pyr): Pregnant female mice were injected solely with pyrazole (100 mg/kg) on days D10 and D13.
✓ Group 3 (C): Control pregnant female mice received an equal volume of 0.9% sodium chloride solution (NaCl 0.9%).

All solutions used were sterilized 10 min before each use, and injections were performed between 10 a.m. and 12 a.m.

**FIGURE 1:**
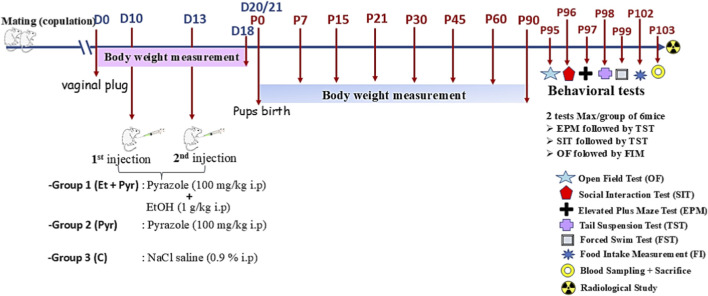
Experimental design and timeline schedule of the experiments.

A total of 18 offspring pups (1 pup from each pregnant dam of the three groups to avoid the litter effect) were included for each study (6 mice of each group used for max two behavioral tests). While tests combination was carried out carefully to avoid a potential interference or habituation effects. All experiments were performed in male pups, with sex subsequently confirmed at adulthood stage (Figure 1).

### 2.4 Biometric and morphometric parameters

#### 2.4.1 Body-weight monitoring

Body weight variation was monitored during two stages:

✓ During the gestational period: pregnant females were monitored at the D0 (positive vaginal pug), D5, D10 (1st injection), D13 (2nd injection), D15 and D18.
✓ During postnatal period: offspring pups were monitored at P7, P15, P21, P30, P45, P60 and P90.

#### 2.4.2 Monitoring the evolution of body size

Body length was measured from the nose to the base of the tail at regular intervals throughout the first 3 months after birth. A graduated ruler was used to ensure precise measurements. Data collection was conducted on postnatal days P0, P7, P15, P21, P30, P45, P60, P90.

#### 2.4.3 Morphometric analysis of vital organs

After sacrifice, the liver, brain, kidneys, and spleen are dissected for morphometric analysis. Each organ was carefully washed and weighed using an analytical balance. The weight of each organ from the animals was then recorded. Then, the ratio of organ weight to body weight was calculated as follows:

$$Organ\;ratio=\frac{\text{Organ}\;\text{weight}\;\left(g\right)}{\text{Body}\;\text{weight}\;\text{at}\;\text{the}\;\text{time}\;\text{of}\;\text{sacrifice}\;\left(g\right)}\times 100$$

#### 2.4.4 Radiological study

The study was on adult (3 months) mice treated as described previously. Before the radiological examination, mice were profoundly anesthetized with urethane (1 g/kg) to be held in specific positions. Radiographic images were taken at the radiology department of Ibn Tofail Private Marrakech hospital, by a specialized technician to enable an analysis of the mice’s skeleton with particular focus on eight distinct bones: femur, tibia, humerus, radius, dorsal rachis, caudal rachis, bi-parietal width, and condyle-basal.

**FIGURE 2:**
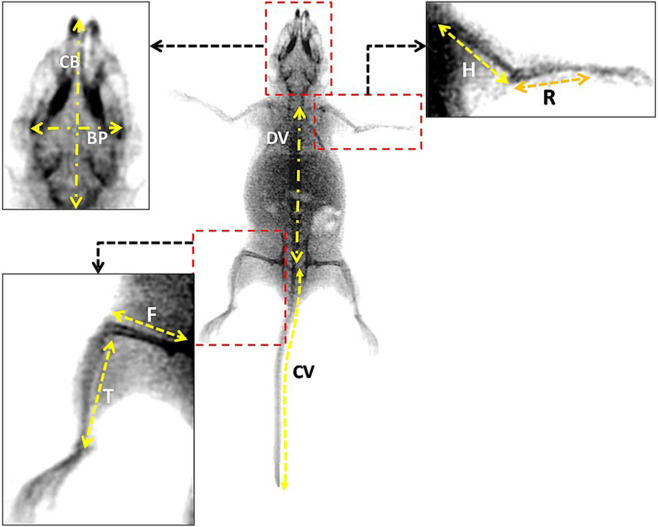
The different measures on the mouse skeleton bones represented on a real X-Rays image on adult mice. (BP) Bi-Parietal width; (CB) Condylobasal; (F) Femur; (H) Humerus; (R) Radius; (DV) Dorsal Vertebrae; (CV) Caudal vertebrae; (T) Tibia.

The following bones measures from each animal of each group were presented in the results section:

- Femur length, tibia (cm), humerus length (cm), radius length (cm), condylobasal length (cm), bi-parietal width length (cm), dorsal rachis length (cm), caudal rachis length (cm).
- For the degree of scoliosis, we measured the angle from the medial line
- For the cranial bones deformations, we measured the hemi-distance of each lateral frontal bone to the medial line (cm) (Figure 2).

### 2.5 Food intake measurement

Each mouse from each experimental group was individually housed for three consecutive days prior to testing to allow habituation to social isolation. Each mouse receives 15 g portions of barley, and its nocturnal food intake was measured over a 12-h period (from 8 p.m. to 8 a.m.) after the remaining food has been dried under a fume hood to eliminate any traces of urine or water, thus reducing the risk of overestimating weight. Therefore, food intake to body weight ratio was calculated for each group (Asakawa et al., 2003).

### 2.6 Neurobehavioral study

A series of neurobehavioral tests were carried out to study the possible anxiety-like state and depressive-like behaviors in our PAE mice. Mice were subjected to handling methods before starting the experiments for at least three consecutive days, with 1 min for each mouse, to reduce the stress levels and fear responses of the experimenter. On the test day, animals were placed in the experimentation room for 30 min of acclimatization before starting the experiment. Finally, the various devices were cleaned after each test with 70% ethanol.

#### 2.6.1 Open field test

Used primarily to measure motor functions, the open field can also be used to assess anxiety-like level. An anxious-like animal avoids the center zone of the open field and stays close to the walls (peripheral area). The time spent in the peripheral area and the number of entries into the central zone are used to assess the degree of anxiety-like in rodents (Seibenhener and Wooten, 2015; El Hiba et al., 2012).

#### 2.6.2 Social interaction test

In this test, we used the open field as an experimental device. Firstly, animals were habituated to the experimental apparatus, where each mouse was placed in the open field for a period of 5 min. Next, an unfamiliar juvenile male mouse was introduced into the same open field, positioned as far away as possible from the mouse under study. Juveniles were carefully selected to minimize confounding variables that could affect social interaction and, thus, ensure reliable results. The selected juvenile mice were 1 month old, an age at which mice are sufficiently developed to interact socially. They are of the same sex as the adult mice tested to prevent undesirable sexual behavior and of standardized size and weight to limit experimental variations. In addition, their health status is checked to ensure they show no signs of infection or other conditions. These juveniles come from the same strain as the adults, which reduces behavioral differences linked to genetic or environmental factors, and they have never been in contact with the adult mice tested, thus ensuring the absence of any previous influence. The behavior of each animal was then recorded using a top-mounted camera for a total of 5 min. Observations recorded included the time spent by the studied mouse engaging in social interactions such as sniffing, nibbling, licking, pushing, jumping on, and going over or under the conspecific, as well as non-social behaviors such as resting, distancing, grooming, and exploring the open field (Swain and Le, 1998). The percentage of time spent engaging in social behaviors and non-social interactions was calculated (Flagstad et al., 2004).

#### 2.6.3 Elevated plus maze test

The experimental setup consists of two open and two closed arms facing each other in the shape of a cross. All raised 50 cm off the ground. Each arm is 30 cm long and 7 cm wide, and 30 cm high walls enclose the closed arms. The test is based on the opposition between the natural behavior of exploring a new environment (the device) and the fear of bright and open environments (open arms). As a natural behavioral tendency, mice preferentially spend more time in the closed arms than in the open arms. However, increased anxiety is reflected by a greater amount of time spent in the closed arms and a higher frequency of closed-arm entries compared with less anxious mice. Following the method described by Benammi et al. (2014), each animal was placed in the center of the elevated plus maze, facing a closed arm. The animals’ behavior was recorded by a camera (Benammi et al., 2014). The time spent in the open arms was quantified and used as an indication of anxiety-like behavior. An arm entry was considered complete only when all four paws were placed inside the arm.

#### 2.6.4 Forced swim test

To assess depression-like, the forced swim test was used. It is one of the most commonly used tests for the study of depressive-like behavior in rodents. It is based on the hypothesis that placing an animal in a container filled with water will initially make efforts to escape but will eventually display an immobility that can be considered a measure of behavioral despair (Porsolt et al., 1977; Petit-Demouliere et al., 2005). This test has been widely used, as it involves exposing animals to stress, which has been shown to play a role in the tendency toward major depression (Porsolt et al., 1978; Cryan et al., 2002). The test is performed in a transparent Plexiglas cylinder (diameter: 20 cm, height: 40 cm) containing 30 cm of water maintained at a temperature of 24 °C ± 1 °C. Mice are habituated 24 h before the test by being placed individually in the water-filled cylinder for 15 min. On test day, each mouse was again placed in the water-filled cylinder. The total immobility and mobility times over a 5-min period were measured (the mouse was considered immobile if it floated in the water, in an upright position, and only made small movements to keep its head above water) (Walf and Frye, 2007).

#### 2.6.5 Tail suspension

We also used the tail suspension test to assess behavioral despair, or depression-like behavior in mice (Duman, 2010). This test is based on the observation that when mice are subjected to short, unavoidable stress by suspending them by their tails, they adopt an immobile posture (Carr and Lucki, 2010). In this test, mice are suspended from a lever by their tail, and their behavior is recorded over a 6-min period. Initially, mice struggle to escape but eventually adopt an immobile posture. The level of immobility relative to the mouse’s active movement is recorded (Salehpour et al., 2019). The duration of both immobility and mobility are measured as an indicator of behavioral despair.

### 2.7 Biochemical assessment

#### 2.7.1 Determination of biochemical markers of liver and kidney function

Serum levels of various markers were measured in samples from the jugular vein of mice in each group under anesthesia (urethane 1 g/kg, i. p.). Blood samples were then centrifuged at 3,000 rpm for 20 min. Aspartate aminotransferase (AST), alanine aminotransferase (ALT), direct bilirubin (BD) and indirect bilirubin (BI), total bilirubin (BT) (as indicative of hepatic function), C-reactive protein (as a marker of systemic inflammation), urea (Ur), and creatinine (Cr) (as indicative of renal function) were measured using an automat (COBAS INTEGRA 400 plus, Laboratory of Biochemistry, Faculty of Medicine and Pharmacy, Cadi Ayyad University, Marrakech, Morocco).

This protocol is based on standard methods widely described in the literature for assessing liver and kidney toxicity in rodents (Dharnidharka et al., 2002; Gomez-Lechon et al., 2003; Chan et al., 2002). These biochemical parameters enable a global assessment of the effect of treatments on target organs.

#### 2.7.2 Assessment of brain oxidative stress

In order to evaluate the possible oxidative stress in the brain of PAE mice, we quantified the activity of the enzymatic biomarker glutathione-S-transferase (GST), catalase (CAT), acetylcholinesterase (AChE), and malondialdehyde (MDA). Briefly, the mice brains are quickly removed and sampled separately at low temperature. All procedures and preparations were performed at 4 °C, then ground with an Ultra-Turax T25 in Tris buffer (100 mM, pH: 7) at a rate of 3 volumes per gram of fresh weight. The homogenate is then centrifuged at 9,000 *g* for 30 min at 4 °C. Of note, to avoid the influence of weight on biomarkers, specimens were previously depicted, and those with the same tissue-weight range were selected throughout the sampling, and the resulting fraction called the S9 fraction (product tissue homogenate; post-mitochondrial fraction) was frozen in Eppendorf tubes at −80 °C until use. The enzymatic biomarker responses, GST, CAT, and AChE, were quantified spectrophotometrically at wavelengths of 340 nm, 240 nm, and 420 nm according to the methods of Habig et al. (1974), Aebi and Bergmeyer, (1983), Ellman et al. (1961) respectively (Habig et al., 1974; Aebi and Bergmeyer, 1983; Ellman et al., 1961). MDA was measured according to the method of Sunderman et al. (1985), which uses the quantification of the formation of TBARS (Thiobarbituric acid reactive substance assay) by reference to MDA absorbance at 530 nm (Sunderman et al., 1985). Total protein in the S9 fraction was determined according to the method of Lowry et al. (1951) using bovine serum albumin (BSA) as standard material (Lowry et al., 1951).

### 2.8 Data acquisition and analysis

To avoid personal influences on the data validity, prior each data analysis including behavioral testing, radiograph scoring, and biochemical assays, specific labeling was assigned to each animal of each group of each study and then, data were analyzed by double-blinded investigators from our team, and the resulting data sets were subsequently compared and reconciled.

### 2.9 Statistical analysis

Statistical analysis was performed using Sigma Stat software for all experiments. Data are expressed as mean ± standard error of the mean (SEM), and a p-value <0.05 was considered statistically significant. Before any analysis, assumptions of normality were checked for each dataset using the Shapiro-Wilk test. Homogeneity of variances (homoscedasticity) was also tested using Levene’s test, in accordance with ANOVA requirements. For the majority of comparisons between treatment groups, one-way ANOVA was used, followed by Tukey’s post-hoc test in case of significance. This choice was based on our initial experimental design, which compared groups according to a single factor (treatment).

## 3 Results

### 3.1 Litter size and postnatal mortality

By calculating the average number of litters per female, we observed a slight but non-significant reduction in litter size in the Et + Pyr group compared with the control (p = 0.620) and Pyr (p = 0.165) groups (Table 1). A similar pattern was observed for postnatal deaths, with a minor, non-significant increase in the average number of postnatally dead offspring per female in the Et + Pyr group compared with the control (p = 0.699) and Pyr (p = 0.699) groups. Additionally, no significant differences were observed between the Pyr and control groups in either litter size (p = 0.699) or postnatal deaths (p = 0.1).

**TABLE 1:** Litter size and postnatal mortality rate in the Et + Pyr females compared to controls and Pyr groups. Data are represented as main value ±SEM.

Groups	Average number of litters per female	Average number of postnatal mortality of fetuses per female
Control group (C)	5.33 ± 0.33	0.33 ± 0.21
Group (Et + Pyr)	5 ± 0.33	0.6 ± 0.36
Pyrazole group (Pyr)	5.5 ± 0.22	0.33 ± 0.21

### 3.2 Body weight evolution of the pregnant females

**FIGURE 3:**
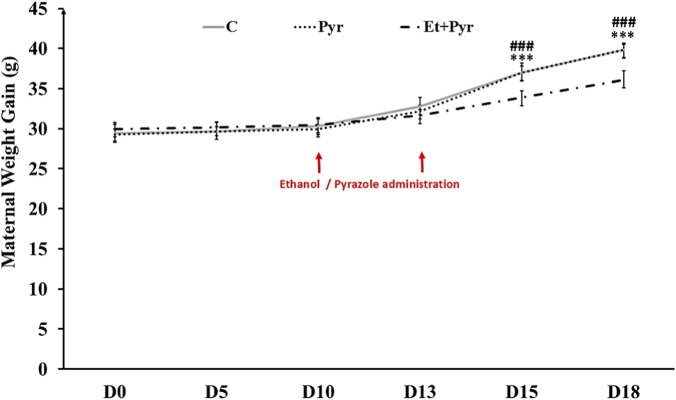
Histograms showing maternal weight gain evolution measurement during gestation in the different studied groups. C (n = 6): control, Pyr (n = 6): mice treated with pyrazole, Et + Pyr (n = 6): group treated with ethanol and pyrazole. Data are presented as mean values ±S.E.M.

Our results showed a slight and non-significant reduction in body weight in Et + Pyr females compared with the Pyr and control groups from D0 to D13; however, highly significant differences were observed at D15 and D18 (p < 0.001; Figure 3).

### 3.3 Body weight, size evolution, and food intake measurement

**FIGURE 4:**
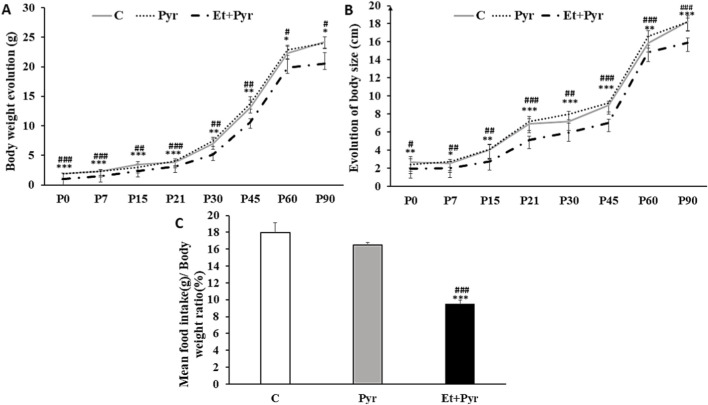
Histograms showing body weight **(A)** and body size **(B)** evolution as well as food intake **(C)** measurement in the different studied groups. C (n = 6): control, Pyr (n = 6): mice treated with pyrazole, Et + Pyr (n = 6): group treated with ethanol and pyrazole. Data are presented as mean values ±S.E.M. *p < 0.05, **p < 0.01, ***p < 0.001 vs. C, #p < 0.05, ##p < 0.01, ###p < 0.001 vs. Pyr.

Our results showed a highly significant decrease in body weight in the Et + Pyr group compared to the control (C) and pyrazole (Pyr) groups from P0 to P90 (Figure 4A). A similar finding was noted for body size, which was significantly reduced in the Et + Pyr group compared to the C and Pyr groups from P7 to P90 (Figure 4B), while no significant difference was found between the C and Pyr groups. Additionally, food intake was significantly decreased in the Et + Pyr group compared to controls (p < 0.001) (Figure 4C). No significant differences were observed between the C and Pyr groups across these parameters.

### 3.4 Morphometric study of vital organs

**FIGURE 5:**
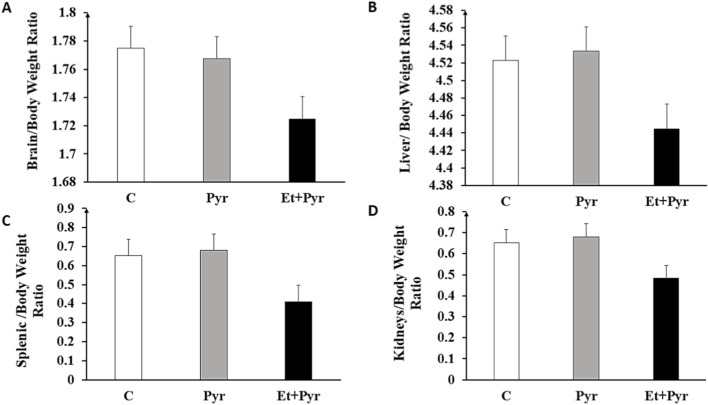
Histogram showing organ-to-bodyweight ratio in the different groups studied. C (n = 6): control, Pyr (n = 6): mice treated with pyrazole, Et + Pyr (n = 6): group treated with ethanol and pyrazole. **(A)** brain, **(B)** liver, **(C)** spleen, **(D)** kidneys. Data are presented as mean values ±S.E.M.

Macroscopically, the external appearance of vital organs, including the brain (Figure 5A), liver (Figure 5B), spleen (Figure 5C), and kidneys (Figure 5D) of Et + Pyr group is similar to that of mice in the C group and Pyr mice. Nevertheless, a slight decrease in organ weight was observed in the Et + Pyr compared with the C and Pyr groups, although this was not statistically significant.

### 3.5 Radiological study

**FIGURE 6:**
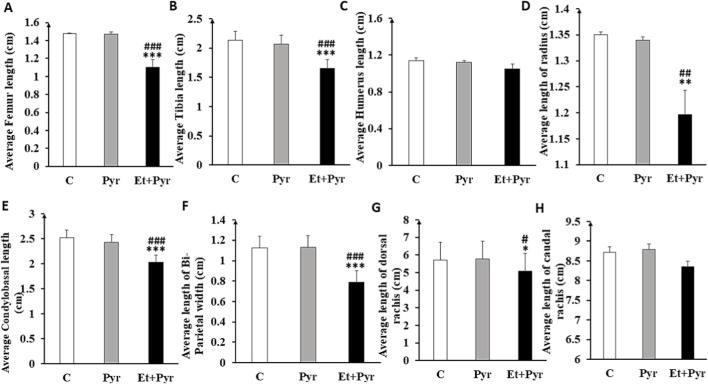
Histograms depicting bones’ lengths in the different studied groups. C (n = 6): control, Pyr (n = 6): mice treated with pyrazole, Et + Pyr (n = 6): group treated with ethanol and pyrazole. **(A)** Average femur length, **(B)** Average tibia length, **(C)** Average humerus length, **(D)** Average radius length, **(E)** Average condylobasal length of the skull, **(F)** Average biparietal width, **(G)** Average dorsal rachis length, **(H)** Average caudal rachis length. Data are presented as mean values ±S.E.M. *p < 0.05, **p < 0.01, ***p < 0.001 vs. C, #p < 0.01, ###p < 0.001 vs. Pyr.

The results show, regarding femur and tibia length, that the Et + Pyr group has significantly lower sizes compared to controls and pyrazole groups (p < 0.001, Figure 6A and p < 0.001; Figure 6B, respectively). Similarly, the foreleg bones reveal significant changes. Hence, the length of the humerus in the Et + Pyr group was slightly reduced compared to the C and Pyr groups, however, this difference was not statistically significant (Figure 6C). For the radius, a statistically highly significant reduction (p < 0.01, Figure 6D) of the bone size was observed in the Et + Pyr group compared with the other two groups. Otherwise, for the caudal and dorsal rachis, we noted the similar trend, with the length of the dorsal rachis being statistically lower (p < 0.05, Figure 6G) in the Et + Pyr group compared with the two other groups, while the caudal rachis did not reach the signification level (Figure 6H; p = 0.397). Finally, our results showed a decrease in condylobasal length and bi-parietal width, this difference being statistically highly significant (p < 0.001, Figure 6E and p < 0.001; Figure 6F, respectively) in the Et + Pyr group compared with Pyr and control mice.

**FIGURE 7:**
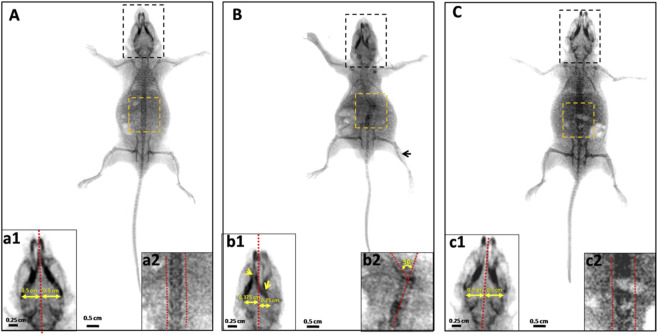
X-ray image of the skeleton of mice from different groups. **(A)** control, **(B)** ethanol + Pyrazole, **(C)** Pyrazole. Black arrow; tibial alteration. b1; cranial asymmetry (yellow arrow). b2: scoliotic deformities.

In addition, a scoliotic deformity is characterized by an abnormal lateral curvature of the rachis (50° to the medial line) which is clearly identifiable in Et + Pyr mice (Figure 7B b2). Whereas, a right asymmetrical cranial bones deformities were also observed with frontal bone dissymmetry in the Et + Pyr (0.25 cm right of the medial line and 0.375 cm left) (Figure 7B b1) compared to controls and Pyr mice (0.5 cm both left and right sides) (Figure 7A a1, Figure 7C c1).

### 3.6 Results of neurobehavioral assessment

#### 3.6.1 Social interaction test

**FIGURE 8:**
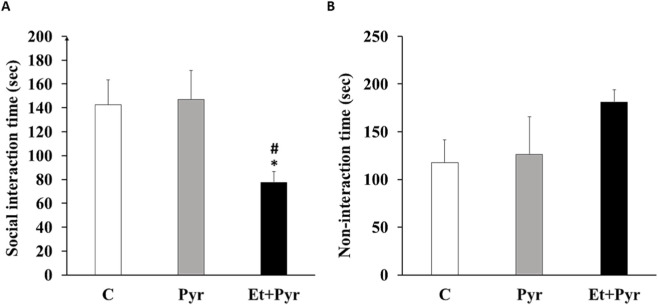
Histograms depicting the average time spent in social interaction **(A)** and non-social interaction **(B)** in the three studied groups. C (n = 6): control, Pyr (n = 6): mice treated with pyrazole, Et + Pyr (n = 6): group treated with ethanol and pyrazole. Data are presented as mean values ±S.E.M. *p < 0.05, #p < 0.05 vs. control.

The effect of PAE on the social behavior of adult mice showed a significant decrease in social interaction time in Et + Pyr group compared to the C and Pyr groups (p < 0.05, Figure 8A). While no significant difference in social interaction time was observed between the controls and Pyr groups (p = 0.855). While non-interaction time exhibited no significant differences between Et + Pyr and controls and Pyr groups (Figure 8B).

#### 3.6.2 Elevated plus maze test

**FIGURE 9:**
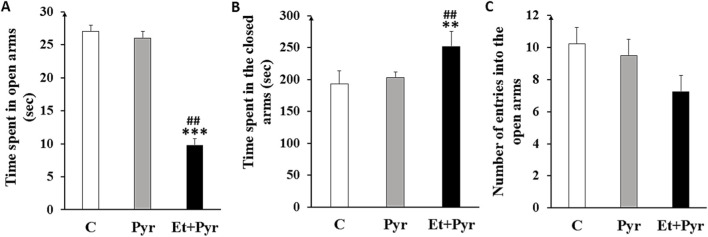
Histograms showing the average time spent in the open arms **(A)**, closed arms **(B)** and number of entries into the open arms **(C)** in the different groups. C (n = 6): control, Pyr (n = 6): mice treated with pyrazole, Et + Pyr (n = 6): group treated with ethanol and pyrazole. Data are presented as mean values ±S.E.M. **p < 0.01, ***p < 0.001 vs. C, ##p < 0.01vs. Pyr.

Anxiety was assessed in our mice using the elevated plus maze test. We observed a highly significant decrease in the time spent in the open arms with a significant increase in time spent closed arms in mice Et + Pyr group compared with the control group, and with mice Pyr group (Figure 9). While no significant differences were noted between the C and Pyr groups.

#### 3.6.3 Open field test

**FIGURE 10:**
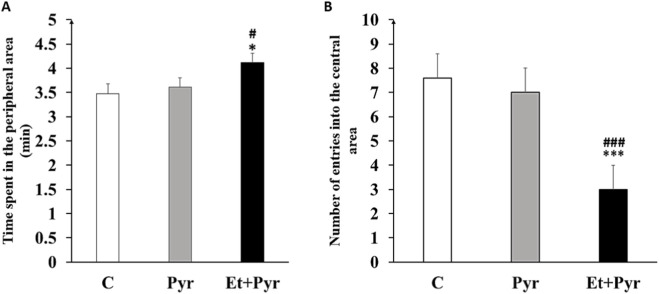
Histograms showing the average time spent in the peripheral area of the open field **(A)** and the number of entries into the central area **(B)** in the different groups. (n = 6). *p < 0.05, ***p < 0.001 vs. C, #p < 0.05, ###p < 0.001 vs. Pyr.

Our results showed a significant increase in the time spent in the peripheral area in Et + Pyr mice compared with C and Pyr groups (p < 0.05, Figure 10A). Otherwise, there was a highly decreased number of entries into the central zone of the open field in Et + Pyr group mice compared with C and Pyr mice (p < 0.001, Figure 10B). However, no difference was observed between the pyrazole and control groups (p = 0.525).

#### 3.6.4 Forced swim and tail suspension tests

**FIGURE 11:**
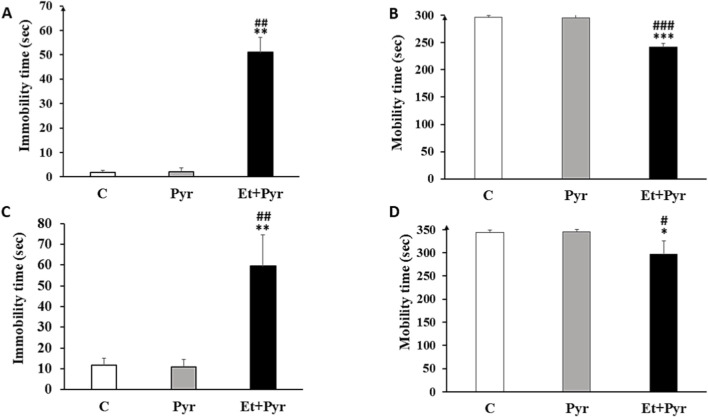
Histograms depicting average immobility and mobility times in the forced swim test and the tail suspension test in the different groups. **(A,B)** Forced swim test; **(C,D)** Tail suspension test. C (n = 6): control, Pyr (n = 6): mice treated with pyrazole, Et + Pyr (n = 6): group treated with ethanol and pyrazole. Data are presented as mean values ±S.E.M. *p < 0.05, **p < 0.01, ***p < 0.01 vs. C, #p < 0.05, ##p < 0.01, ###p < 0.001 vs. Pyr.

Our results showed a highly significant increase in immobility time (Figure 11A) with decreased mobility (Figure 11B) in the Et + Pyr group compared with C and Pyr groups in the forced-swim test. Similarly, in the tail suspension test, Et + Pyr group exhibited a significant increase in immobility time (Figure 11C) and a decrease in mobility (Figure 11D) compared to both the C and Pyr groups. However, no significant difference between the Pyr group and the C group in both tests was noted.

### 3.7 Biochemical analysis

#### 3.7.1 Study of liver and kidney functions

**FIGURE 12:**
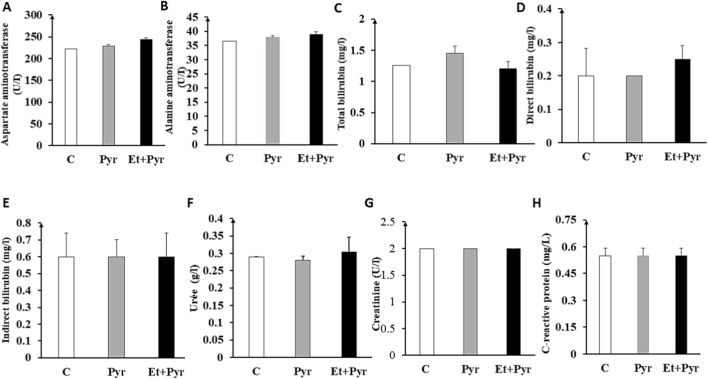
Histograms depicting plasma levels of different biochemical markers of liver and kidney functions. **(A)** Aspartate aminotransferase, **(B)** Alanine aminotransferase, **(C)** Total bilirubin, **(D)** Direct bilirubin, **(E)** Indirect bilirubin, **(F)** Urea, **(G)** Creatinine, **(H)** C-reactive protein in the studied groups: C (n = 5): control, Pyr (n = 5): mice treated with pyrazole, Et + Pyr (n = 5): group treated with ethanol and pyrazole. Data are presented as mean values ±S.E.M.

Our results of biochemical parameters of liver and kidney functions in the different groups show subtle but not significant variations in all the assessed markers. Uremia appears to be slightly increased in mice treated with Et + Pyr group compared to the C groups and to the Pyr group, but these differences are not statistically significant (p = 0.242; p = 0.486, respectively, Figure 12). Likewise, creatinine levels did not show significant variations between the groups (Figure 12). Regarding the liver enzymes AST and ALT, a slight increase, but not significant, is observed in Et + Pyr group compared to the other two groups: C (p = 0.07; p = 0.960, respectively) and Pyr (p = 0.096; p = 0.288, respectively).

Additionally, direct, indirect, and total bilirubin as well as CRP levels did not show any statistically significant differences among the three studied groups (Figure 12).

#### 3.7.2 Brain oxidative stress

**FIGURE 13:**
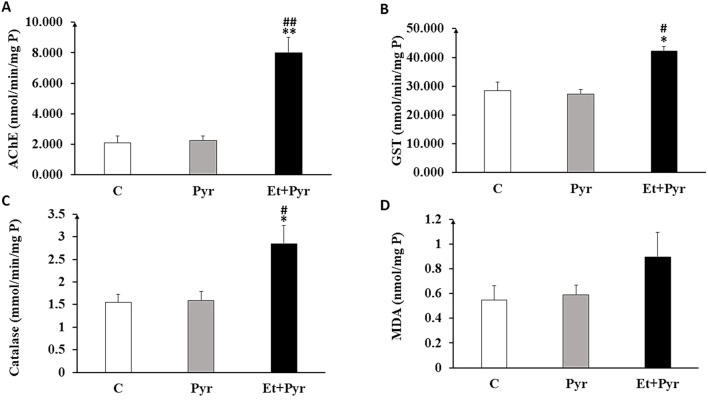
Histograms depicting brain tissue levels of oxidative stress markers **(A)** acetylcholinesterase (AChE). **(B)** Glutathione S-Transferase (GST). **(C)** catalase (CAT). **(D)** malondialdehyde (MDA) at the brain level in the different groups studied. C (n = 5): control, Pyr (n = 5): mice treated with pyrazole, Et + Pyr (n = 5): mice treated with ethanol + pyrazole. Data are presented as mean values ±S.E.M. ∗ p < 0.05, **p < 0.01 vs. C, #p < 0.05, ##p < 0.01 vs. Pyr.

Our results of the possible state of brain oxidative stress following PAE showed a significant elevation in AChE activity in the Et + Pyr group compared to the C and mice Pyr (p < 0.01, Figure 13A). Similarly, there was a significant increase in the GST (p < 0.05, Figure 13B), and catalase activities (p < 0.05, Figure 13C), in the Et + Pyr group compared to C and mice Pyr group. However, there was no difference between the C group and Pyr mice. The measurement of MDA levels, as a product of lipid peroxidation, shows a slight non-significant increase in the Et + Pyr group compared to controls and Pyr mice (p = 0.09; p = 0.1, respectively; Figure 13D).

## 4 Discussion

Through the present study, we explored the consequences of PAE on postnatal development in mice, with a focus on mood disorders, particularly depression and anxiety-like states, along with body growth patterns. Female mice with vaginal plugs received two intraperitoneal injections of ethanol (1 g/kg BW) at the GD10 and GD13 concomitant with pyrazole, which is used to inhibit the enzyme alcohol dehydrogenase (AD) leading to prolonged ethanol half-life. Indeed, according to previous literature reports on rats and mice, blood ethanol immediately increases following intraperitoneal administration and is maintained over 6 h (Livy et al., 2003). With pyrazole (used in the present investigation), body clearance of ethanol may be slowed allowing ethanol to exert its central effects. This increases the direct exposure of the developing brain to alcohol, enabling a better understanding of the direct effects of ethanol on the nervous system during development while minimizing the influence of metabolites such as acetaldehyde toxicity (Smimih et al., 2023).

Here, we have brought further data on the neurotoxic effects of ethanol exposure during the early life stages on the adult CNS function. Our results showed, the presence of anxiety and depressive-like behaviors in mice exposed prenatally to alcohol, as well as a severe deficiency in whole-body growth. At the peripheral level, our PAE mice exhibited a notable decrease in body weight concomitant with body growth deficits, even observed in the skeleton size of different bones. These results highlight the teratogenic potential of PAE, widely recognized for its ability to disrupt normal fetal development and compromise intrauterine growth. More specifically, the decrease in body weight observed in offspring is consistent with a large body of data showing that PAE can significantly alter somatic growth trajectories. Several experimental studies in rodents have demonstrated that exposure to ethanol during gestation results in a marked reduction in birth weight, as well as postnatal growth retardation (Smimih et al., 2023; Carter et al., 2012; Chen and Nyomba, 2003; Day et al., 2002). These alterations could result from alcohol-induced placental dysfunction, increased oxidative stress, and impaired nutrient transport, all of which contribute to growth restriction during critical developmental periods. Thus, in pregnant mice intoxicated orally with 25% (v/v) ethanol, some authors have reported a decrease in body weight at birth and at the adult stage reflecting marked teratogenic effects (Abbott et al., 2016). This type of exposure mimics regular, sustained alcohol consumption by pregnant women. However, other studies found no significant effect of PAE on body weight or postnatal growth, which could be explained by differences in exposure protocol. For example, some studies have used low to moderate doses (below 2 g/kg/day), often administered over limited time windows (*e.g*., GD7 to GD9) or intermittent modes of administration, which do not reflect chronic intoxication conditions (Kaminen-Ahola et al., 2010; Ieraci and Herrera, 2006). These parameters can significantly influence the occurrence or non-occurrence of teratogenic effects, underlining the importance of dose, duration, and mode of exposure in the interpretation of results. Indeed, (Middaugh et al., 1988), using the C57BL/6Cr mouse model, reported that the body weight of pups born to females fed a liquid diet containing 5% ethanol (v/v) throughout gestation did not differ significantly from that of controls until weaning. This protocol, based on continuous moderate chronic exposure, could explain the lack of effect on growth observed in this study (Middaugh et al., 1988).

Similarly, some studies have explored the molecular mechanisms underlying the effects of PAE on body weight which may involve a possible altered IGF-A and two and/or negative growth regulators H9 expressions (Marjonen et al., 2018; Martín-Estal et al., 2022).

On the other hand, our PAE mice exhibited decreased food intake, suggesting that PAE could disrupt the regulation of appetite and food intake or even profoundly affect energy metabolism. Indeed, it has been suggested that alcohol and its metabolites may interfere with neuroendocrine pathways regulating hunger and satiety, which could explain this reduction in food intake (Wurst et al., 2007; Calissendorff et al., 2006). Moreover, our results are consistent with those described by (Montagud-Romero et al., 2021), who examined the impact of PAE on food intake in adolescent mice. The study found that mice exposed to alcohol consumed significantly less food compared to control mice (Montagud-Romero et al., 2021). However, in another study, authors examined the long-term consequences of PAE on metabolic functions, including food intake, in adult mice and found that mice exposed to alcohol during gestation showed impairments in food intake, increased appetite, and higher food consumption compared to control mice (Calissendorff et al., 2006). However, other studies have reported no significant alterations in food consumption in mice prenatally exposed to alcohol (Chaudoin et al., 2023). These discrepancies can be attributed to variations in experimental protocols, exposure duration and period, and the animal model used.

Such effects on food intake behavior could imply a possible neuro-modulatory effect of alcohol. Whereas, our study revealed skeletal morphological abnormalities, suggesting that the effects of prenatal alcohol exposure may extend well beyond the digestive and metabolic systems, also affect bone development. In particular, a scoliotic deformity, characterized by an abnormal lateral curvature of the spine, is clearly identifiable in mice treated with ethanol. In addition, alterations were observed in the tibia, suggesting significant structural changes in this region. Additionally, we noted cranial deformities (asymmetry), indicating disturbances in skull bone formation. Our results support observations from other studies indicating that PAE can significantly impact body growth and bone development (Wu et al., 2020). Alternatively, the reduction in bone length still observed in our mice highlights the negative effect of alcohol on bone mineralization and density, probably due to disturbances in calcium homeostasis and dysfunctions of osteoblasts, essential for bone growth (Snow and Keiver, 2007). A study conducted by (Snow and Keiver, 2007) investigating rats at the fetal stage revealed that prenatal exposure to ethanol results in a reduction in fetal bone length, attributed to shortening of the shaft and a decrease in the length of the resting zone (Snow and Keiver, 2007). Furthermore, ethanol appears to induce subtle changes in the organization of the epiphysis, including an increase in the length of the hypertrophic zone. These results suggest that ethanol affects later stages of bone development, probably after cartilage mineralization, while its impact on the resting zone indicates that it could also influence the early stages of the developmental process. In another study of children diagnosed with FASD or at risk for FASD, it was shown that during adolescence (10–15 years), subjects with FASD were significantly smaller and had lower bone mineral density, bone mineral content, and lean tissue mass (Young et al., 2022).

We also evaluated the weight of vital organs; although we did not observe significant macroscopic modifications, a trend toward reduction in the weight of these organs without apparent changes in their morphology was observed, suggesting a subtle effect of alcohol exposure on organ growth and functions. These observations are in agreement with previous studies, which highlighted the direct cytotoxic effects of alcohol, capable of inducing oxidative stress and contributing to organ atrophy (Weeks et al., 2020). Besides, biochemical analyses revealed no significant differences in markers of hepatic and renal function, including notably urea, creatinine, AST, ALT, and bilirubin, as well as the inflammatory marker C-reactive protein (CRP) in PAE mice leading to exclude subsequent liver and/or kidneys dysfunctions. However, our data appear to be inconsistent with other studies reporting biochemical variations in markers of renal and hepatic function following exposure to ethanol (Akison et al., 2020; Nakhoul et al., 2017; Liu et al., 2016). Such a discrepancy would probably be due to differences between our dose used and those of other studies, which may be below the threshold of toxicity manifested, the period of exposure, and even the animal species used. Indeed, a study carried out by (Rozman et al., 2010) highlighted the importance of the dose in the manifestation of the toxic effects of ethanol (Rozman et al., 2010). On the other hand, at the central level, our results revealed significant changes in the oxidative stress markers in the brain tissue of mice exposed to ethanol, indicating a potential brain oxidative stress following PAE. Hence, the increase in AChE activity in mice exposed to ethanol could have detrimental effects on the cholinergic neurotransmission in the brain of PAE mice, suggesting the potential effect of alcohol on the cholinergic system. Whereas, we noted a particular discrepancy with regards to previous studies reporting inhibition rather than increased AChE activities, both of them may reflect either a possible fetal imbalance in the cholinergic neurotransmission patterns or even a neurotoxic effect through promoting increased ROS production and decreased antioxidant capacity, leading to oxidative stress and aggravating cellular damage in the brain (Costa et al., 2013). Furthermore, the significant increase in GST activity in ethanol-exposed mice suggests an increased antioxidant response to ROS in the brain following PAE. Such an increase is consistent with previous studies that have also demonstrated an increase in GST in response to oxidative stress induced by prenatal exposure to ethanol or other toxic compounds (Montoliu et al., 1995; Devi et al., 1996; Shearn et al., 2016).

In addition, the enzymatic activity of catalase also showed a significant increase in mice exposed to ethanol, indicating, as for the previous enzymes, an antioxidant response to oxidative stress. It is important to note that approximately 60% of the enzymatic system involved in ethanol metabolism is already active during early ontogeny (Zimatkin et al., 2006; Aragon et al., 1991), with peak activity occurring from late gestation to the first postnatal week (Del Maestro and McDonald, 1987). These developmental dynamics may partly explain the observed increase in catalase activity in response to ethanol-induced oxidative stress during this sensitive period. This observation is consistent with other studies that also reported an increase in catalase in response to ethanol-induced oxidative stress (Shili et al., 2021; D’aloisio et al., 2022). Additionally, MDA levels showed a slight and non-significant increase in mice prenatally exposed to ethanol. However, previous studies have reported a significant increase in MDA levels in response to ethanol exposure, suggesting significant lipid peroxidation and cellular damage (Lopatynska-Mazurek et al., 2021; Tsermpini et al., 2022; Gauthier et al., 2005). While MDA did not reach statistical significance, we observed significant increases in AChE activity, Catalase, and GST, indicating activation of antioxidant defense mechanisms. These changes reflect altered redox homeostasis and a compensatory response to increased oxidative burden, even in the absence of robust lipid peroxidation.

Our neurobehavioral study revealed elevated anxiety and depressive-like behaviors as well as impaired social interaction in PAE mice. Such disturbances could be attributed to dysregulation of brain structures involved in the control of anxiety, depression, and social behavior, including the prefrontal cortex, hippocampus, amygdala, and nucleus accumbens. These regions form an integrated neural network that regulates emotional processing, stress responsiveness, and social cognition, and are particularly vulnerable to prenatal environmental insults including PAE. Disruption of their structural and functional connectivity during critical periods of neurodevelopment may therefore underlie the behavioral abnormalities observed in PAE mice. Our finding is corroborated by studies conducted by (Fish et al., 2016), who reported altered social behavior and altered sociability in adult mice prenatally exposed to alcohol (Fish et al., 2016). Additionally, in rats exposed to alcohol, Sliwowska et al. (2010) found a reduction in the hippocampal volume, a structure involved in the regulation of anxiety (Sliwowska et al., 2010). Thus (Sliwowska et al., 2010), demonstrated that prenatal exposure to alcohol leads to the dysregulation of the hypothalamic-pituitary-adrenal axis in mice (Wieczorek et al., 2015). This axis is responsible for controlling the body’s response to stress, and its dysregulation can contribute to anxiety-like responses. In another study conducted by Marquardt and Brigman (2016), the prefrontal cortex and the amygdala, two critical brain regions involved in the regulation of social behavior were examined. The authors noted a significant decrease in neuronal density in the prefrontal cortex of mice exposed to alcohol compared to control mice (Marquardt and Brigman, 2016). Furthermore, the alcohol-exposed group showed reduced expression of genes associated with synaptic plasticity and amygdala neurodevelopment, a key structure in anxiety control and social behavior. Another study by Laufer et al. (2013) explored the role of epigenetic modifications in prenatal alcohol-induced social deficits in mice and noted alterations in the DNA methylation patterns of specific genes linked to social behavior in mice exposed to alcohol (Laufer et al., 2013). Otherwise, previous reports have examined the impact of prenatal exposure to alcohol on the hippocampus and prefrontal cortex, structures involved in mood control. They observed a significant reduction in hippocampal volume in adults prenatally exposed to alcohol compared to controls, indicating structural alterations associated with prenatal alcohol exposure (Coles et al., 2011). Additionally, previous reports attempted to explore the molecular mechanisms involved in the development of depressive-like behaviors following prenatal alcohol exposure. Indeed (Yu et al., 2020), showed that prenatal exposure to ethanol in Wistar rats led to notable alterations within the hippocampus, including decreased levels of the neurotrophic factor BDNF, changes in mRNA expression of genes associated with glucocorticoid receptors and synaptic plasticity, as well as upregulation of genes related to apoptosis (Yu et al., 2020).

## 5 Conclusion

In summary, our study demonstrated that ethanol exposure during early life stages (pregnancy) can lead to the onset of anxiety- and depressive-like behaviors, as well as a severe deficiency in whole-body growth, including body weight and the skeleton size of different bones. While our study provided insightful evidence of the PAE outcomes on the adulthood stage, the exclusive use of male in our study may present certain limitation, however, it may raise the question on the disagree of females responsiveness similarity to males which needs further investigations. On the other hand, a possible interference between locomotor deficits and the anxious and depressive-like states and the limited number of tests used (2 per behavioral pattern), may represent further implicit and unavoidable limitation that should be taken into account when considering these findings.

## Data Availability

The original contributions presented in the study are included in the article/supplementary material, further inquiries can be directed to the corresponding authors.

## References

[B1] Abbaoui A. El Hiba O. Gamrani H. (2016). Copper poisoning induces neurobehavioral features of Parkinson’s disease in rat: alters dopaminergic system and locomotor performance. Park. Relat. *Disord*. 22, e188. 10.1016/j.parkreldis.2016.02.047

[B2] Abbott C. W. Kozanian O. O. Kanaan J. Wendel K. M. Huffman K. J. (2016). The impact of prenatal ethanol exposure on neuroanatomical and behavioral development in mice. Alcohol Clin. Exp. Res. 40, 122–133. 10.1111/acer.12936 26727530 PMC4702517

[B3] Aebi H. E. (1983). “Catalase,”. Methods of enzyme analysis. Editor Bergmeyer H. U. (Weinheim, Germany: Verlag Chemie), 3, 273–285.

[B4] Akison L. K. Probyn M. E. Gray S. P. Cullen-McEwen L. A. Tep K. Steane S. E. (2020). Moderate prenatal ethanol exposure in the rat promotes kidney cell apoptosis, nephron deficits, and sex-specific kidney dysfunction in adult offspring. Anat. Rec. 303, 2632–2645. 10.1002/ar.24370 31984647

[B5] Alfonso-Loeches S. Guerri C. (2011). Molecular and behavioral aspects of the actions of alcohol on the adult and developing brain. Crit. Rev. *Clin. Lab. Sci*. 48, 19–47. 10.3109/10408363.2011.580567 21657944

[B6] Aragon C. M. G. Amit Z. Stotland L. M. (1991). Studies on ethanol–brain catalase interaction: evidence for central ethanol oxidation. Alcohol Clin. Exp. Res. 15, 165–169. 10.1111/j.1530-0277.1991.tb01848.x 2058789

[B7] Asakawa A. Inui A. Kaga T. Katsuura G. Fujimiya M. Fujino M. A. (2003). Antagonism of ghrelin receptor reduces food intake and body weight gain in mice. Gut 52, 947–952. 10.1136/gut.52.7.947 12801949 PMC1773718

[B8] Benammi H. El Hiba O. Romane A. Gamrani H. (2014). A blunted anxiolytic-like effect of curcumin against acute lead-induced anxiety in rat: involvement of serotonin. Acta histochem. 116, 920–925. 10.1016/j.acthis.2014.03.002 24721902

[B9] Benammi H. Erazi H. El Hiba O. Vinay L. Bras H. Viemari J.-C. (2017). Disturbed sensorimotor and electrophysiological patterns in lead intoxicated rats during development are restored by curcumin I. PLoS ONE 12, e0172715. 10.1371/journal.pone.0172715 28267745 PMC5340392

[B10] Brancato A. Castelli V. Cavallaro A. Lavanco G. Plescia F. Cannizzaro C. (2018). Pre-conceptional and peri-gestational maternal binge alcohol drinking produces inheritance of mood disturbances and alcohol vulnerability in the adolescent offspring. Front. Psychiatry 9, 150. 10.3389/fpsyt.2018.00150 29743872 PMC5930268

[B11] Brocardo P. S. Gil-Mohapel J. Christie B. R. (2011). The role of oxidative stress in fetal alcohol spectrum disorders. Brain Res. Rev. 67, 209–225. 10.1016/j.brainresrev.2011.02.001 21315761

[B12] Calissendorff J. Danielsson O. Brismar K. Röjdmark S. (2006). Alcohol ingestion does not affect serum levels of peptide YY but decreases both total and octanoylated ghrelin levels in healthy subjects. Metabolism 55, 1625–1629. 10.1016/j.metabol.2006.08.003 17142135

[B13] Carr G. V. Lucki I. (2010). “The role of serotonin in depression,” in Handbook of behavioral neuroscience (Amsterdam, Netherlands: Elsevier), 493–505.

[B14] Carter R. C. Jacobson J. L. Molteno C. D. Jiang H. Meintjes E. M. Jacobson S. W. (2012). Effects of heavy prenatal alcohol exposure and iron deficiency anemia on child growth and body composition through age 9 years. Alcohol Clin. Exp. Res. 36, 1973–1982. 10.1111/j.1530-0277.2012.01810.x 22897691 PMC3697011

[B15] Cederbaum A. I. (2012). Alcohol metabolism. Clin. Liver Dis. 16, 667–685. 10.1016/j.cld.2012.08.002 23101976 PMC3484320

[B16] Centers for Disease Control and Prevention (2005). Advisory on alcohol use in pregnancy: a 2005 message to women from the US surgeon general. CDC. Available online at: https://stacks.cdc.gov/view/cdc/26056 (accessed on September 24, 2025).

[B17] Chan T. M. Fang G. X. Tang C. S. O. Cheng I. K. P. Lai K. N. Ho S. K. N. (2002). Preemptive lamivudine therapy based on HBV DNA level in HBsAg-positive kidney allograft recipients. Hepatology 36, 1246–1252. 10.1053/jhep.2002.36156 12395336

[B18] Chaudoin T. R. Bonasera S. J. Dunaevsky A. Padmashri R. (2023). Exploring behavioral phenotypes in a mouse model of fetal alcohol spectrum disorders. Dev. *Neurobiol*. 83, 184–204. 10.1002/dneu.22922 37433012 PMC10546278

[B19] Chen L. Nyomba B. L. G. (2003). Effects of prenatal alcohol exposure on glucose tolerance in the rat offspring. Metabolism 52, 454–462. 10.1053/meta.2003.50073 12701058

[B20] Coles C. D. Goldstein F. C. Lynch M. E. Chen X. Kable J. A. Johnson K. C. (2011). Memory and brain volume in adults prenatally exposed to alcohol. Brain Cogn. 75, 67–77. 10.1016/j.bandc.2010.08.013 21067853 PMC3026602

[B21] Costa L. G. Giordano G. Guizzetti M. (2013). Inhibition of cholinergic muscarinic signaling by ethanol: potential mechanism of developmental neurotoxicity and biological plausibility for the beneficial effects of choline supplementation. Int. *J. Alcohol Drug Res*. 2, 17–25. 10.7895/ijadr.v2i3.72

[B22] Cryan J. F. Markou A. Lucki I. (2002). Assessing antidepressant activity in rodents: recent developments and future needs. Trends Pharmacol. Sci. 23, 238–245. 10.1016/s0165-6147(02)02017-5 12008002

[B23] Day N. L. Leech S. L. Richardson G. A. Cornelius M. D. Robles N. Larkby C. (2002). Prenatal alcohol exposure predicts continued deficits in offspring size at 14 years of age. Alcohol Clin. Exp. Res. 26, 1584–1591. 10.1097/01.ALC.0000034036.75248.D9 12394293

[B24] Del Maestro R. McDonald W. (1987). Distribution of superoxide dismutase, glutathione peroxidase and catalase in developing rat brain. Mech. Ageing Dev. 41, 29–38. 10.1016/0047-6374(87)90051-0 3431167

[B25] DeSesso J. M. Scialli A. R. Holson J. F. (1999). Apparent lability of neural tube closure in laboratory animals and humans. Am. J. Med. *Genet*. 87, 143–162. 10.1002/(sici)1096-8628(19991119)87:2<143::aid-ajmg6>3.0.co;2-j 10533029

[B26] Devi B. G. Schenker S. Mazloum B. Henderson G. I. (1996). Ethanol-induced oxidative stress and enzymatic defenses in cultured fetal rat hepatocytes. Alcohol 13, 327–332. 10.1016/0741-8329(96)00002-x 8836319

[B27] Dharnidharka V. R. Kwon C. Stevens G. (2002). Serum cystatin C is superior to serum creatinine as a marker of kidney function: a meta-analysis. Am. J. Kidney Dis. 40, 221–226. 10.1053/ajkd.2002.34487 12148093

[B28] Duman C. H. (2010). Models of depression. Vitam. Horm. 82, 1–21. 10.1016/S0083-6729(10)82001-1 20472130

[B29] D’aloisio G. Acevedo M. B. Angulo-Alcalde A. Trujillo V. Molina J. C. (2022). Moderate ethanol exposure during early ontogeny of the rat alters respiratory plasticity, ultrasonic distress vocalizations, increases brain catalase activity, and acetaldehyde-mediated ethanol intake. Front. Behav. Neurosci. 16, 1031115. 10.3389/fnbeh.2022.1031115 36439967 PMC9686390

[B30] El Hiba O. Gamrani H. Ahboucha S. (2012). Increased Reissner’s fiber material in the subcommissural organ and ventricular area in bile duct ligated rats. Acta Histochem. 114, 673–681. 10.1016/j.acthis.2011.12.002 22209469

[B31] Ellman G. L. Courtney K. D. Andres V. Featherstone R. M. (1961). A new and rapid colorimetric determination of acetylcholinesterase activity. Biochem. Pharmacol. 7, 88–95. 10.1016/0006-2952(61)90145-9 13726518

[B32] Fish E. W. Holloway H. T. Rumple A. Baker L. K. Wieczorek L. A. Moy S. S. (2016). Acute alcohol exposure during neurulation: behavioral and brain structural consequences in adolescent C57BL/6J mice. Behav. Brain Res. 311, 70–80. 10.1016/j.bbr.2016.05.004 27185739 PMC4931949

[B33] Flagstad P. Mørk A. Glenthøj B. Y. Van Beek J. Michael-Titus A. T. Didriksen M. (2004). Disruption of neurogenesis on gestational day 17 in the rat causes behavioral changes relevant to positive and negative schizophrenia symptoms and alters amphetamine-induced dopamine release in nucleus accumbens. Neuropsychopharmacology 29, 2052–2064. 10.1038/sj.npp.1300516 15199377

[B34] Gao Y. Zhou Z. Ren T. Kim S. J. He Y. Seo W. (2019). Alcohol inhibits T-cell glucose metabolism and hepatitis in ALDH2-deficient mice and humans: roles of acetaldehyde and glucocorticoids. Gut 68, 1311–1322. 10.1136/gutjnl-2018-316221 30121625 PMC6582747

[B35] Gauthier T. W. Ping X.-D. Harris F. L. Wong M. Elbahesh H. Brown L. A. S. (2005). Fetal alcohol exposure impairs alveolar macrophage function *via* decreased glutathione availability. Pediatr. Res. 57, 76–81. 10.1203/01.PDR.0000149108.44152.D3 15531743

[B36] Gaztañaga M. Angulo-Alcalde A. Chotro M. G. (2020). Prenatal alcohol exposure as a case of involuntary early onset of alcohol use: consequences and proposed mechanisms from animal studies. Front. Behav. Neurosci. 14, 26. 10.3389/fnbeh.2020.00026 32210773 PMC7066994

[B37] Ghasoub M. Scholten C. Perdue M. Long M. Ostertag C. Kar P. (2025). Associations between white matter asymmetry and communication skills in children with prenatal alcohol exposure. Drug Alcohol Depend. 269, 112674. 10.1016/j.drugalcdep.2025.112674 40311557

[B38] Gil-Mohapel J. Bianco C. D. Cesconetto P. A. Zamoner A. Brocardo P. S. (2019). “Ethanol exposure during development, and brain oxidative stress,” in Neuroscience of alcohol. Editors Watson R. Preedy V. (Cambridge, MA, USA: Elsevier), 493–503. 10.1016/B978-0-12-813125-1.00039-9

[B39] Gomez-Lechon M. J. Donato M. T. Castell J. V. Jover R. (2003). Human hepatocytes as a tool for studying toxicity and drug metabolism. Curr. Drug Metab. 4, 292–312. 10.2174/1389200033489424 12871046

[B40] Graves L. Carson G. Poole N. Patel T. Bigalky J. Green C. R. (2020). Guideline No. 405: screening and counselling for alcohol consumption during pregnancy. J. Obstet. *Gynaecol. Can*. 42, 1158–1173. 10.1016/j.jogc.2020.03.004 32900457

[B41] Habig W. H. Pabst M. J. Jakoby W. B. (1974). Glutathione S-transferases: the first enzymatic step in mercapturic acid formation. J. Biol. Chem. 249, 7130–7139. 10.1016/S0021-9258(19)42083-8 4436300

[B42] Hellemans K. G. C. Sliwowska J. H. Verma P. Weinberg J. (2010). Prenatal alcohol exposure: fetal programming and later life vulnerability to stress, depression and anxiety disorders. Neurosci. Biobehav. Rev. 34, 791–807. 10.1016/j.neubiorev.2009.06.004 19545588 PMC5518679

[B43] Ieraci A. Herrera D. G. (2006). Nicotinamide protects against ethanol-induced apoptotic neurodegeneration in the developing mouse brain. PLoS Med. 3, e101. 10.1371/journal.pmed.0030101 16478293 PMC1370925

[B44] Kaminen-Ahola N. Ahola A. Maga M. Mallitt K.-A. Fahey P. Cox T. C. (2010). Maternal ethanol consumption alters the epigenotype and the phenotype of offspring in a mouse model. PLoS Genet. 6, e1000811. 10.1371/journal.pgen.1000811 20084100 PMC2797299

[B45] Laufer B. I. Mantha K. Kleiber M. L. Diehl E. J. Addison S. M. F. Singh S. M. (2013). Long-lasting alterations to DNA methylation and ncRNAs could underlie the effects of fetal alcohol exposure in mice. Dis. Model. Mech. 6, 977–992. 10.1242/dmm.010975 23580197 PMC3701217

[B46] Lewis P. (2009). Australian guidelines to reduce health risks from drinking alcohol. Canberra, Australia: National Health and Medical Research Council.

[B47] Li X. Weng X. Shi H. Gao R. Wang P. Jia D. (2019). Acetaldehyde dehydrogenase 2 deficiency exacerbates cardiac fibrosis by promoting mobilization and homing of bone marrow fibroblast progenitor cells. J. Mol. Cell Cardiol. 137, 107–118. Epub 2019 Oct 24. PMID: 31668970. 10.1016/j.yjmcc.2019.10.006 31668970

[B48] Liu Q. Gao F. Liu X. Li J. Wang Y. Han J. (2016). Prenatal alcohol exposure and offspring liver dysfunction: a systematic review and meta-analysis. Arch. Gynecol. Obstet. 294, 225–231. 10.1007/s00404-016-4109-7 27168179

[B49] Livy D. J. Parnell S. E. West J. R. (2003). Blood ethanol concentration profiles: a comparison between rats and mice. Alcohol 29 (3), 165–171. 10.1016/s0741-8329(03)00025-9 12798972

[B50] Lopatynska-Mazurek M. Komsta L. Gibula-Tarlowska E. Kotlinska J. H. (2021). Aversive learning deficits and depressive-like behaviors are accompanied by an increase in oxidative stress in a rat model of fetal alcohol spectrum disorders: the protective effect of rapamycin. Int. J. Mol. Sci. 22, 7083. 10.3390/ijms22137083 34209274 PMC8268794

[B51] Lowry O. H. Rosebrough N. J. Farr A. L. Randall R. J. (1951). Protein measurement with the folin phenol reagent. J. Biol. Chem. 193, 265–275. 14907713

[B52] Marjonen H. Toivonen M. Lahti L. Kaminen-Ahola N. (2018). Early prenatal alcohol exposure alters imprinted gene expression in placenta and embryo in a mouse model. PLoS ONE 13, e0197461. 10.1371/journal.pone.0197461 29763474 PMC5953443

[B53] Marquardt K. Brigman J. L. (2016). The impact of prenatal alcohol exposure on social, cognitive and affective behavioral domains: insights from rodent models. Alcohol 51, 1–15. 10.1016/j.alcohol.2015.12.002 26992695 PMC4799836

[B54] Martín-Estal I. Fajardo-Ramírez Ó. R. Bermúdez de León M. Zertuche-Mery C. Benavides-Guajardo R. García-Cruz M. I. (2022). Effect of ethanol consumption on the placenta and liver of partially IGF-1-deficient mice: the role of metabolism via CYP2E1 and the antioxidant enzyme system. Biol. (Basel) 11, 1264. 10.3390/biology11091264 36138743 PMC9495332

[B55] May C. R. Mair F. Finch T. MacFarlane A. Dowrick C. Treweek S. (2009). Development of a theory of implementation and integration: normalization process theory. Implement. Sci. 4, 29. 10.1186/1748-5908-4-29 19460163 PMC2693517

[B56] Middaugh L. D. Randall C. L. Favara J. P. (1988). Prenatal ethanol exposure in C57 mice: effects on pregnancy and offspring development. Neurotoxicol. Teratol. 10, 175–180. 10.1016/0892-0362(88)90082-7 3398826

[B57] Molina J. C. Moyano H. F. Spear L. P. Spear N. E. (1984). Acute alcohol exposure during gestational day 8 in the rat: effects upon physical and behavioral parameters. Alcohol 1, 459–464. 10.1016/0741-8329(84)90036-7 6543578

[B58] Molina J. C. Hoffmann H. Spear L. P. Spear N. E. (1987). Sensorimotor maturation and alcohol responsiveness in rats prenatally exposed to alcohol during gestational day 8. Neurotoxicol. Teratol. 9, 121–128. 10.1016/0892-0362(87)90026-6 3657747

[B59] Montagud-Romero S. Cantacorps L. Fernández-Gómez F. J. Núñez C. Miñarro J. Rodríguez-Arias M. (2021). Unraveling the molecular mechanisms involved in alcohol intake and withdrawal in adolescent mice exposed to alcohol during early life stages. Prog. Neuropsychopharmacol. Biol. Psychiatry 104, 110025. 10.1016/j.pnpbp.2020.110025 32599136

[B60] Montoliu C. Sancho-Tello M. Azorin I. Burgal M. Valles S. Renau-Piqueras J. (1995). Ethanol increases cytochrome P4502E1 and induces oxidative stress in astrocytes. J. Neurochem. 65, 2561–2570. 10.1046/j.1471-4159.1995.65062561.x 7595552

[B61] Nakhoul M. R. Seif K. E. Haddad N. Haddad G. E. (2017). Fetal alcohol exposure: the common toll. J. Alcohol Drug Depend. 5, 1. 10.4172/2329-6488.1000257 28868323 PMC5575798

[B62] Petit-Demouliere B. Chenu F. Bourin M. (2005). Forced swimming test in mice: a review of antidepressant activity. Psychopharmacology 177, 245–255. 10.1007/s00213-004-2048-7 15609067

[B63] Popova S. Lange S. Shield K. Mihic A. Chudley A. E. Mukherjee R. A. S. (2016). Comorbidity of fetal alcohol spectrum disorder: a systematic review and meta-analysis. *Lancet* 387, 978–987. 10.1016/S0140-6736(15)01345-8 26777270

[B64] Popova S. Lange S. Probst C. Gmel G. Rehm J. (2017). Estimation of national, regional, and global prevalence of alcohol use during pregnancy and fetal alcohol syndrome: a systematic review and meta-analysis. Lancet Glob. Health 5, e290–e299. 10.1016/S2214-109X(17)30021-9 28089487

[B65] Popova S. Lange S. Probst C. Gmel G. Rehm J. (2018). Global prevalence of alcohol use and binge drinking during pregnancy, and fetal alcohol spectrum disorder. Biochem. Cell Biol. 96, 237–240. 10.1139/bcb-2017-0077 28834683

[B66] Popova S. Charness M. E. Burd L. Crawford A. Hoyme H. E. Mukherjee R. A. S. (2023a). Fetal alcohol spectrum disorders. Nat. Rev. Dis. Prim. 9, 11. 10.1038/s41572-023-00462-2 36823161

[B67] Popova S. Dozet D. Pandya E. Sanches M. Brower K. Segura L. (2023b). Effectiveness of brief alcohol interventions for pregnant women: a systematic literature review and meta-analysis. BMC Pregnancy Childbirth 23, 61. 10.1186/s12884-023-05355-y 36694121 PMC9872314

[B68] Porsolt R. D. Le Pichon M. Jalfre M. (1977). Depression: a new animal model sensitive to antidepressant treatments. Nature 266, 730–732. 10.1038/266730a0 559941

[B69] Porsolt R. D. Anton G. Blavet N. Jalfre M. (1978). Behavioural despair in rats: a new model sensitive to antidepressant treatments. Eur. J. Pharmacol. 47, 379–391. 10.1016/0014-2999(78)90118-8 204499

[B70] Rice D. Barone S. (2000). Critical periods of vulnerability for the developing nervous system: evidence from humans and animal models. Environ. *Health Perspect*. 108, 511–533. 10.1289/ehp.00108s3511 10852851 PMC1637807

[B71] Riley E. P. Infante M. A. Warren K. R. (2011). Fetal alcohol spectrum disorders: an overview. Neuropsychol. Rev. 21, 73–80. 10.1007/s11065-011-9166-x 21499711 PMC3779274

[B72] Risbud R. D. Breit K. R. Thomas J. D. (2022). Early developmental alcohol exposure alters behavioral outcomes following adolescent re-exposure in a rat model. Alcohol Clin. Exp. Res. 46, 1993–2009. 10.1111/acer.14993 36117379 PMC9722643

[B73] Rozman K. K. Doull J. Hayes W. J. (2010). “Dose and time determining, and other factors influencing, toxicity,” in Hayes’ handbook of pesticide toxicology Editor Krieger R. 3rd ed. (San Diego, CA, USA: Elsevier), 3–101.

[B74] Rungratanawanich W. Lin Y. Wang X. Kawamoto T. Chidambaram S. B. Song B. J. (2023). ALDH2 deficiency increases susceptibility to binge alcohol-induced gut leakiness, endotoxemia, and acute liver injury in mice through the gut-liver axis. Redox Biol. 59, 102577. Epub 2022 Dec 13. PMID: 36528936; PMCID: PMC9792909. 10.1016/j.redox.2022.102577 36528936 PMC9792909

[B75] Salehpour F. Farajdokht F. Cassano P. Sadigh-Eteghad S. Erfani M. Hamblin M. R. (2019). Near-infrared photobiomodulation combined with coenzyme Q10 for depression in a mouse model of restraint stress: reduction in oxidative stress, neuroinflammation, and apoptosis. Brain Res. Bull. 144, 213–222. 10.1016/j.brainresbull.2018.10.010 30385146 PMC6309497

[B76] Seibenhener M. L. Wooten M. C. (2015). Use of the open field maze to measure locomotor and anxiety-like behavior in mice. J. Vis. Exp. 96, e52434. 10.3791/52434 25742564 PMC4354627

[B77] Shearn C. T. Fritz K. S. Shearn A. H. Saba L. M. Mercer K. E. Engi B. (2016). Deletion of GSTA4-4 results in increased mitochondrial post-translational modification of proteins by reactive aldehydes following chronic ethanol consumption in mice. Redox Biol. 7, 68–77. 10.1016/j.redox.2015.11.013 26654979 PMC4683459

[B78] Shili I. Hamdi Y. Marouani A. Lasfar Z. B. Ghrairi T. Lefranc B. (2021). Long-term protective effect of PACAP in a fetal alcohol syndrome (FAS) model. Peptides 146, 170630. 10.1016/j.peptides.2021.170630 34481915

[B79] Sliwowska J. H. Barker J. M. Barha C. K. Lan N. Weinberg J. Galea L. A. M. (2010). Stress-induced suppression of hippocampal neurogenesis in adult male rats is altered by prenatal ethanol exposure. Stress 13, 302–314. 10.3109/10253890903531582 20536332 PMC4833451

[B80] Smimih K. El-Mansoury B. Saad F.E.-Z. Khanouchi M. El Amine S. Aimrane A. (2023). Sensory motor function disturbances in mice prenatally exposed to low dose of ethanol: a neurobehavioral study in postnatal and adult stages. Neurol. Int. 15, 580–594. 10.3390/neurolint15020580 37092508 PMC10123635

[B81] Snow M. E. Keiver K. (2007). Prenatal ethanol exposure disrupts the histological stages of fetal bone development. Bone2007 41, 181–187. 10.1016/j.bone.2007.04.182 17532282 PMC2039868

[B82] Sulik K. K. Johnston M. C. Webb M. A. (1981). Fetal alcohol syndrome: embryogenesis in a mouse model. Science 214, 936–938. 10.1126/science.6795717 6795717

[B83] Sunderman F. W. Marzouk A. Hopfer S. M. Zaharia O. Reid M. C. (1985). Increased lipid peroxidation in tissues of nickel chloride-treated rats. Ann. Clin. Lab. Sci. 15, 229–236. 3994292

[B84] Swain M. G. Le T. (1998). Chronic cholestasis in rats induces anhedonia and a loss of social interest. Hepatology 28, 6–10. 10.1002/hep.510280102 9657089

[B85] Tadokoro T. Oura K. Nakahara M. Fujita K. Tani J. Morishita A. (2025). Genetic polymorphisms of ALDH2 and ADH1B in alcohol-induced liver injury: molecular mechanisms of inflammation and disease progression in East Asian populations. Int. J. Mol. Sci. 26 (17), 8328. 10.3390/ijms26178328 40943250 PMC12428050

[B86] Tsermpini E. E. PlemenitašIlješ A. Dolžan V. (2022). Alcohol-induced oxidative stress and the role of antioxidants in alcohol use disorder: a systematic review. Antioxidants 11, 1374. 10.3390/antiox11071374 35883865 PMC9311529

[B87] Ukita K. Fukui Y. Shiota K. (1993). Effects of prenatal alcohol exposure in mice: influence of an ADH inhibitor and a chronic inhalation study. Reprod. *Toxicol*. 7, 273–281. 10.1016/0890-6238(93)90234-x 8318759

[B88] Walf A. A. Frye C. A. (2007). The use of the elevated plus maze as an assay of anxiety-related behavior in rodents. Nat. Protoc. 2, 322–328. 10.1038/nprot.2007.44 17406592 PMC3623971

[B89] Weeks O. Bossé G. D. Oderberg I. M. Akle S. Houvras Y. Wrighton P. J. (2020). Fetal alcohol spectrum disorder predisposes to metabolic abnormalities in adulthood. J. Clin. Investig. 130, 2252–2269. 10.1172/JCI132139 32202514 PMC7190939

[B90] Weinberg J. Sliwowska J. H. Lan N. Hellemans K. G. C. (2008). Prenatal alcohol exposure: foetal programming, the hypothalamic–pituitary–adrenal axis and sex differences in outcome. J. *Neuroendocrinol*. 20, 470–488. 10.1111/j.1365-2826.2008.01669.x 18266938 PMC8942074

[B91] Wieczorek L. Fish E. W. O’Leary-Moore S. K. Parnell S. E. Sulik K. K. (2015). Hypothalamic-pituitary-adrenal axis and behavioral dysfunction following early binge-like prenatal alcohol exposure in mice. Alcohol 49, 207–217. 10.1016/j.alcohol.2015.01.005 25709101 PMC4414725

[B92] World Health Organization (2014). Guidelines for identification and management of substance use and substance use disorders in pregnancy. Switzerland: WHO. 24783312

[B93] Wu Z. Pan Z. Wen Y. Xiao H. Shangguan Y. Wang H. (2020). Egr1/p300/ACE signal mediates postnatal osteopenia in female rat offspring induced by prenatal ethanol exposure. Food Chem. *Toxicol*. 136, 111083. 10.1016/j.fct.2019.111083 31887396

[B94] Wurst F. M. Rasmussen D. D. Hillemacher T. Kraus T. Ramskogler K. Lesch O. (2007). Alcoholism, craving, and hormones: the role of leptin, ghrelin, prolactin, and the pro-opiomelanocortin system in modulating ethanol intake. Alcohol Clin. Exp. Res. 31, 1963–1967. 10.1111/j.1530-0277.2007.00531.x 18034691

[B95] Young S. L. Gallo L. A. Brookes D. S. K. Hayes N. Maloney M. Liddle K. (2022). Altered bone and body composition in children and adolescents with confirmed prenatal alcohol exposure. Bone 164, 116510. 10.1016/j.bone.2022.116510 35931325

[B96] Yu Y. Shi Z. Xu D. Li Y. Qin J. Zhang Z. (2020). Prenatal ethanol exposure increases susceptibility to depression- and anxiety-like behavior in adult female offspring and its underlying mechanism. Reprod. *Toxicol*. 96, 36–46. 10.1016/j.reprotox.2020.05.015 32497709

[B97] Zahid K. R. Yao S. Khan A. R. R. Raza U. Gou D. (2019). mTOR/HDAC1 crosstalk mediated suppression of ADH1A and ALDH2 links alcohol metabolism to hepatocellular carcinoma onset and progression *in silico* . Front. Oncol. 9, 1000. 10.3389/fonc.2019.01000 31637215 PMC6787164

[B98] Zimatkin S. M. Pronko S. P. Vasiliou V. Gonzalez F. J. Deitrich R. A. (2006). Enzymatic mechanisms of ethanol oxidation in the brain. Alcohol Clin. Exp. Res. 30, 1500–1505. 10.1111/j.1530-0277.2006.00181.x 16930212

